# House dust mites use a plant-like siRNA pathway to silence transposable elements

**DOI:** 10.1371/journal.pgen.1007255

**Published:** 2018-04-05

**Authors:** Lichao Li, Weifeng Gu

**Affiliations:** Department of Molecular, Cell and Systems Biology, University of California, Riverside, California, United States of America; Cornell University, UNITED STATES

Evidence from the past decade has established that RNA interference (RNAi) plays versatile and important roles in regulating gene expression in all domains of life [1, 2]. There are three major classes of small RNAs, including microRNAs (miRNAs), short interfering RNAs (siRNAs), and Piwi-interacting RNAs (piRNAs). miRNAs primarily regulate functional genes and play important roles in development and other processes, while siRNAs and piRNAs primarily regulate nonfunctional transcripts, serving as defense mechanisms against transposable elements (TEs) and viruses [3]. Plants do not have piRNAs and use siRNAs made by Dicer to silence TEs [4]. Animals usually have siRNAs and piRNAs, both of which can be used to silence TEs. E.g., in vertebrates and flies, piRNAs play major roles in silencing TEs [5]. *Caenorhabditis elegans* uses secondary siRNAs made by RNA-dependent RNA polymerases (RdRP)—not by Dicer—to silence TEs [6]. However, at least in the Tc3 transposon, a conserved family of DNA transposons, secondary siRNAs are initiated by piRNAs in *C*. *elegans* [7]. Regardless, piRNAs in animals are either directly or indirectly involved in silencing TEs. In this issue, Mondal and Flynt reported house dust mites do not possess any piRNA pathway [8]. To maintain genome stability, they use Dicer-dependent siRNAs to silence TEs. This finding puts house dust mites in a special position in the evolution of small RNA pathways, bearing a plant-like system for silencing TEs in an animal system. Indeed, the size of siRNAs in house dust mites is similar to that made by plant Dicer-like 3 (DCL3) [9], which is also involved in silencing transposons. This research further diversifies the functions of animal small RNAs and demonstrates a unified TE-regulation mechanism in plants and animals.

## Distinct biogenesis pathways of piRNAs

**Fig 1:**
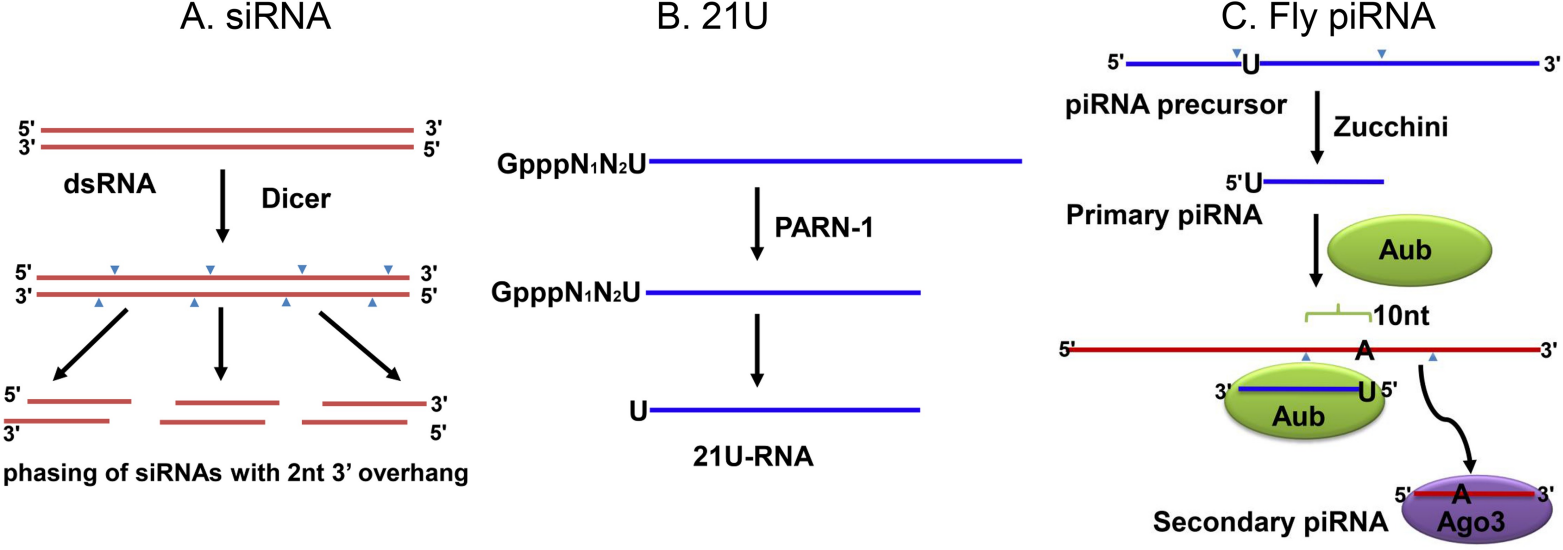
The biogenesis of siRNAs and piRNAs. A. siRNAs are generated processively from dsRNAs in a Dicer-dependent manner. B. *C*. *elegans* 21Us are generated from csRNAs in a Dicer-independent but PARN-1–dependent manner. C. The Dicer-independent ping-pong model for piRNA biogenesis in flies. csRNA, capped small RNA; dsRNA, double-stranded RNA; piRNA, Piwi-interacting RNA; siRNA, short interfering RNA.

Both miRNAs and siRNAs are generated by Dicer using double-stranded RNAs (dsRNAs). Since Dicer cleaves long dsRNAs in a processive manner and generates 2-nt 3′ overhangs, product siRNAs, a.k.a. primary siRNAs, display a phasing pattern with a 2-nt overlap between the 3′ ends of sense and antisense siRNAs (Fig 1A). In contrast, piRNAs are made in Dicer-independent manners. In *C*. *elegans*, the piRNAs, 21Us (21 nt with 5′ U), are made from short single-stranded RNAs (ssRNAs), also called capped small RNAs (csRNAs) [10]. PARN-1, a 3′ to 5′ exonuclease, is responsible for removing the extra nucleotides from the 3′ end of csRNAs [11] (Fig 1B). In *Drosophila*, piRNAs are 24–30 nts long, usually with 5′ U and composed of primary and secondary piRNAs [12]. Primary piRNAs are originally processed from ssRNAs by the endonuclease Zucchini, while secondary piRNAs are made by a conserved ping-pong mechanism, using a Piwi/primary piRNA complex to cleave targets, generating secondary piRNAs from targets [12]. Thus, primary and secondary piRNAs overlap 10 nts at 5′ ends, and secondary piRNAs usually have an “A” at the 10th position, base-paring with the 5′ U of primary piRNAs (Fig 1C).

## TEs are silenced using distinct pathways in plants and animals

In vertebrates, piRNAs play critical roles in silencing TEs in male germlines. In invertebrates, piRNAs may or may not directly silence TEs. E.g., piRNAs in many insects are directly involved in silencing TEs. By contrast, 21Us in *C*. *elegans* are not directly involved in silencing transposons. Instead, secondary siRNAs (22Gs) made by nonprocessive RdRPs, which make short RNAs, play TE-silencing roles. However, at least one 21U is required for silencing Tc3 indirectly by initiating 22Gs, and the rest is considered as a reservoir for recognizing and silencing nonself genes via initiating 22Gs [5,7]. In some chelicerae arthropods, such as *Dermatophagoides farinae*, piRNAs are involved in silencing TEs. However, in other chelicerae arthropods, such as scabies mite, it seems that there is no piRNA pathway. Plants do not have piRNAs and use siRNAs primarily made by DCL3 to silence TEs [9].

## House dust mites do not have a functional piRNA pathway

Mondal and Flynt [8] constructed a more complete house dust mite genome and transcriptome using high-throughput sequencing. Using bioinformatics, they identified three Dicer alleles (DfaDcr1–3) and eight Argonautes (DfaAgo1–8). None of these Argonauts belongs to the Piwi clade, which binds piRNAs. Also missing is a functional *Hen1* gene, which is able to modify/stabilize piRNAs. These facts suggest that there is no piRNA pathway in house dust mites. Small RNA sequencing results corroborated this, since: (1) there is no 5′ U preference, a piRNA sequence feature; (2) there is no “A” preference at position 10, as expected from ping-pong piRNAs; and (3) the 3′ end of small RNAs does not have 2′-O-methylation, another feature of piRNAs.

## House dust mites express TE-silencing siRNAs

The house dust mite small RNAs demonstrate typical siRNA features generated by Dicer, since they: (1) are dependent on Dicer; (2) have 5′ monophosphate and unmodified 3′ end; and (3) display a 2-nt overlapping pattern between siRNAs derived from opposite strands of dsRNAs. As expected, the TE-derived and Dicer-dependent siRNAs are required for silencing TEs since the knockdown of Dicer desilences TEs.

## Unanswered questions about the siRNA-mediated TE silencing

Although the piRNA result is based on negative data, the authors did provide both bioinformatical and experimental data to cross-examine the conclusion. More importantly, they showed siRNAs substitute for piRNAs in silencing TEs. The size of these siRNAs, approximately 24 nts, is interesting since they are not similar to that of siRNAs in animals but to that of plants. Although *C*. *elegans* also uses siRNAs to silence TEs, those siRNAs are made only by nonprocessive RdRPs, while house dust mite siRNAs are made by Dicer using long dsRNA synthesized by processive RdRPs. Among DfaDcr1–3, only DfaDcr1 is processive. However, all three Dicers are more or less required for the siRNA biogenesis. Do these Dicers generate different groups of siRNAs? Among the eight Argonautes, do they bind different groups of siRNAs (primary or secondary)/miRNAs? Since the small RNAs only displayed one peak (24 nt), are miRNAs in this organism also 24 nts rather than 22–23 nts long? If so, this organism seems to use a special mode to process and load small RNAs, including miRNAs. The lack of 5-methylcytosine (m^5^C) modification on house dust mite genome may suggest histone modifications may play more important roles in epigenetic regulations. Do these 24-nt siRNAs mediate histone modifications?
